# К юбилею главного редактора журнала «Проблемы эндокринологии» академика РАН Ивана Ивановича Дедова

**DOI:** 10.14341/probl12728

**Published:** 2021-01-23

**Keywords:** Иван Иванович Дедов, эндокринология, здравоохранение

## Abstract

12 февраля 2021 г. отмечает юбилей академик РАН, выдающийся ученый с мировым именем Иван Иванович Дедов. Иван Иванович - ведущий клиницист эндокринолог, педагог, опытный организатор здравоохранения России. И. И. Дедов возглавил отечественную эндокринологию на сложном этапе ее развития. Под его руководством клиническая эндокринология, узкая, во многом второстепенная область, приобрела в настоящее время значение фундаментальной и приоритетной клинической дисциплины.

Редакционная коллегия журнала «Проблемы эндокринологии» сердечно поздравляет своего главного редактора и желает ему здоровья и долгих плодотворных лет на благо отечественной эндокринологии.

## Abstract (en)

On February 12, 2021, Ivan I. Dedov, Academician of the Russian Academy of Sciences, an outstanding scientist with a worldwide reputation, celebrates his anniversary. Ivan I. Dedov is a leading clinician, endocrinologist, teacher, experienced organizer of healthcare in Russia. Ivan I. Dedov headed the Russian endocrinology at a difficult stage of its development. Under his leadership, clinical endocrinology, a narrow, largely secondary field, has now acquired the significance of a fundamental and priority clinical discipline.

The editorial board of the journal «Problems of Endocrinology» cordially congratulates its editor-in-chief and wishes him good health and many fruitful years for the benefit of Russian endocrinology.

**Figure:**
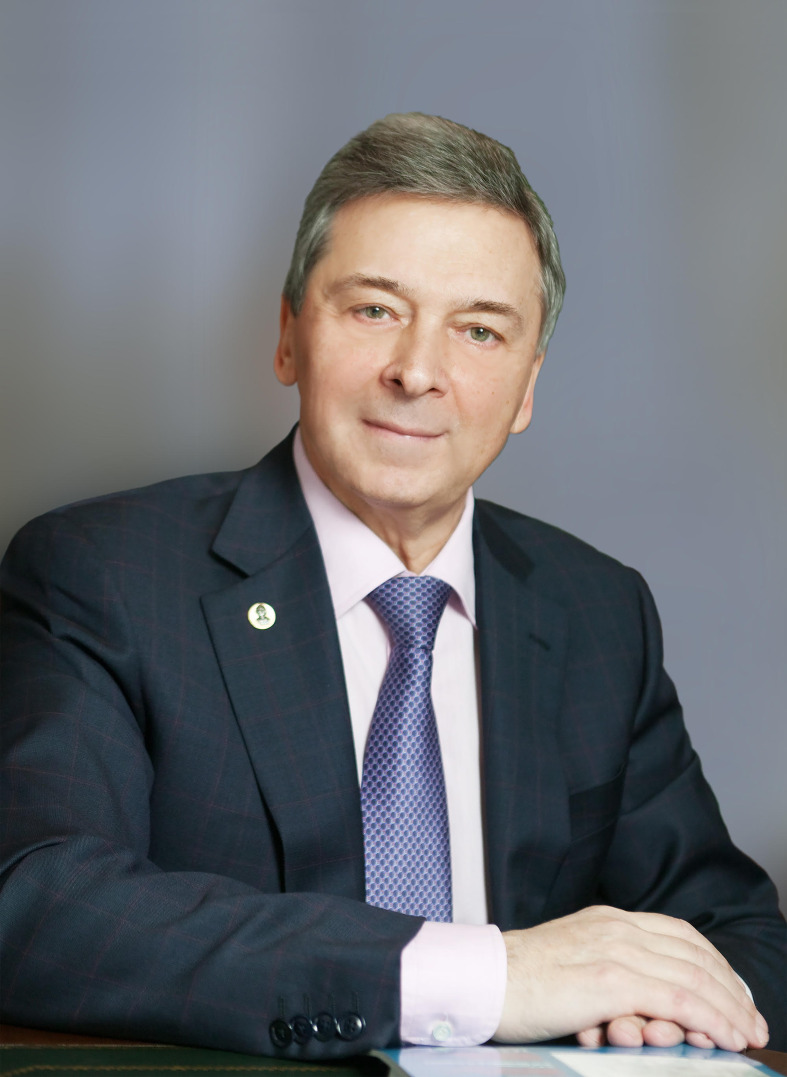


12 февраля 2021 г. отмечает юбилей академик РАН, выдающийся ученый с мировым именем Иван Иванович Дедов — ведущий клиницист-эндокринолог, педагог, опытный организатор здравоохранения России.

И.И. Дедов родился в Воронежской области, в селе Дмитряшевка Хлевенского района Липецкой области. Окончив в 1958 г. среднюю школу с золотой медалью, он поступает в Воронежский медицинский институт.

С 1964 г. И.И. Дедов работает в качестве младшего научного сотрудника лаборатории нейроэндокринологии и группы эндокринологии Института медицинской радиологии АМН СССР в Обнинске. В 1973 г. И.И. Дедова приглашают в Москву, и до 1982 г. он работает старшим научным сотрудником лаборатории экспериментальной эндокринологии Института экспериментальной и клинической онкологии АМН СССР. Докторскую диссертацию защитил в 1976 г. С 1982 по 1988 гг. — профессор кафедры факультетской терапии 1-го лечебного факультета Первого МГМУ им. И.М. Сеченова, а с 1988 г. — заведующий кафедрой эндокринологии того же Института.

Дедовым И.И. создан единственный в России и уникальный в мировой медицине ФГБУ «Эндокринологический национальный центр» (ЭНЦ), объединяющий 7 научно-исследовательских институтов: Институт диабета, Институт клинической эндокринологии, Институт детской эндокринологии, Институт репродуктивной эндокринологии, Институт персонализированной эндокринологии, Институт онкоэндокринологии, Институт образовательной деятельности. С 1989 г. по 2019 г. И.И. Дедов являлся директором ЭНЦ — единственного в России и широко известного за ее пределами научно-исследовательского, клинико-диагностического, лечебного, организационно-методического и педагогического комплекса эндокринологического профиля. С 2019 г. по настоящий момент И.И. Дедов является президентом ФГБУ «НМИЦ эндокринологии» Минздрава России.

ЭНЦ участвует в реализации Национального проекта «Здравоохранение», в том числе по направлениям «Демография», «Детство», «Онкология»: профилактика и лечение эндокринологических, сердечно-сосудистых, онкологических, включая широкий спектр орфанных и таких социально значимых заболеваний, как сахарный диабет, метаболический синдром, ожирение.

Ежегодно в клиниках Центра более 70 000 россиян получают высококвалифицированную специализированную лечебную помощь.

В рамках приоритетного Национального проекта «Здоровье» Дедовым И.И. была реализована научно обоснованная и экономически просчитанная программа, основанная на «Национальных стандартах» развития специализированной и высокотехнологичной медицинской помощи (ВМП) по эндокринологии в Российской Федерации. Эта программа до настоящего времени позволяет тиражировать высокие медицинские технологии, объединяет первичное звено, муниципальные и региональные (межрегиональные) уровни и федеральный центр в единую систему; создает реальные условия доступности ВМП для населения всех, без исключения, регионов России, играет ключевую роль в народосбережении и консолидации усилий по модернизации здравоохранения.

Академик И.И. Дедов является координатором Национальной программы «Борьба с йододефицитными заболеваниями щитовидной железы», в рамках которой проводятся работы по мониторингу йододефицитных состояний в Российской Федерации, а также анализируется влияние ключевых зобогенных и антропогенных факторов на состояние здоровья населения страны с реализацией популяционной, групповой и индивидуальной профилактики болезней щитовидной железы. Результаты исследований легли в основу постановления Правительства Российской Федерации и Национальной программы «О мерах профилактики заболеваний, связанных с дефицитом йода».

По инициативе и при непосредственном участии И.И. Дедова с 1996 г. была разработана и реализована Федеральная целевая программа (ФЦП) «Сахарный диабет», с 2002 г. входившая в ФЦП «Предупреждение и борьба с социально значимыми заболеваниями». В соответствии с резолюциями ООН и ВОЗ, определившими сахарный диабет как опаснейший вызов мировому сообществу, в рамках программы была модернизирована современная диабетологическая служба России, кардинально изменившая ситуацию в стране и позволившая России войти в первую десятку стран мира по качеству и доступности специализированной помощи многим миллионам больных сахарным диабетом.

Впервые в России создан и эффективно функционирует Государственный регистр больных сахарным диабетом, который стал уникальной информационно-аналитической системой диабетологической службы России. Сегодня в регионах РФ функционируют более 130 диабетологических центров и диспансеров, 1900 школ по обучению больных взрослых, детей и их родителей, свыше 180 референс-отделений по лечению диабетической ретино- и нефропатии, диабетической стопы. По инициативе И.И. Дедова впервые в России разработаны мобильные лечебно-диагностические комплексы («Диамобили»), которые позволяют оказывать доступную и высококлассную специализированную медицинскую помощь сельскому населению и жителям отдаленных регионов Российской Федерации.

К фундаментальным работам мирового уровня И.И. Дедова относятся многолетние исследования по генетике, иммуногенетике и гормонально-метаболическим маркерам сахарного диабета. Под его руководством открыты гаплотипы, определяющие как индивидуальные риски, так и риски заболеть сахарным диабетом в этнически разных группах населения Российской Федерации. Эти уникальные данные вошли в Международный реестр иммуногенетических исследований сахарного диабета и позволили организовать в России сеть медико-генетических консультаций для прогноза и мониторинга здоровья в группах риска, что позволило рассчитать финансово-экономические, организационные и социальные составляющие для практического здравоохранения регионов РФ.

Под руководством И.И. Дедова разработаны и внедрены полные инновационные цепочки от геномных проектов до новейших технологий в области диагностики, лечения и профилактики таких социально значимых болезней эндокринной системы, определяющих медицинскую составляющую демографической ситуации в России, как сахарный диабет, болезни репродуктивной системы и щитовидной железы, опухоли эндокринной системы. Создан целый ряд панелей генов, каждая из которых рассчитана на прицельное, в зависимости от клинической ситуации, секвенирование генов, обеспечивая создание генетического паспорта отдельного человека, семьи, этноса. Врач получил возможность предсказывать и нивелировать риски заболеваний, их раннее выявление и назначать максимально эффективную персонализированную терапию.

В ФГБУ «НМИЦ эндокринологии» Минздрава России под руководством И.И. Дедова реализуется эндокринологическая составляющая национальной программы «Здоровый ребенок», в рамках которой впервые в России проводится неонатальный скрининг 3 орфанных заболеваний:

В 2015 г. введен в эксплуатацию новый корпус Института детской эндокринологии «НМИЦ эндокринологии», в котором созданы и успешно функционируют новые клинико-лабораторные подразделения, отделение вспомогательных репродуктивных технологий, клинические отделения по всему профилю детской эндокринологии, отделение радионуклидной диагностики и терапии, биобанки раритетных биоматериалов, лаборатория клэмп-технологий и персонализированной терапии сахарного диабета, отделения наследственных и орфанных заболеваний, постнатального мониторинга наследственных эндокринопатий, отдел геномных, транскриптомных, протеомных и метаболомных технологий, отдел патоморфологии с лабораторией электронной микроскопии.

Дедовым И.И. разработана принципиально новая доктрина профилактической (предсказательной) эндокринологии, основанная на геномных, постгеномных, иммунных, гормонально-метаболических и клеточных технологиях. Персонализированная модель эндокринологии позволяет прогнозировать риски болезней и их осложнений, а клинические, молекулярно-генетические и гормонально-метаболические технологии позволяют подобрать лечение персонально каждому больному, а не лечить по шаблону болезнь. Результаты этих исследований, охватывающие ключевые направления современной эндокринологии, диабетологии, онкоэндокринологии, репродуктивной эндокринологии, легли в основу рекомендаций, по которым организована работа эндокринологической службы Российской Федерации.

Впервые в истории, благодаря работе уникального отделения вспомогательных репродуктивных технологий, созданного И.И. Дедовым в «НМИЦ эндокринологии», с помощью предимплантационных технологий появилась возможность у людей с наследственными заболеваниями, желающими иметь ребенка, исключить из этапа оплодотворения «больную клетку», несущую ген болезни, и тем самым «прервать» многовековые цепочки наследственных заболеваний и подарить таким семьям счастье рождения здорового ребенка.

Дедов И.И., являясь руководителем головного Центра по проблеме «Эндокринология», главным специалистом-экспертом эндокринологом и председателем профильной комиссии по эндокринологии Министерства здравоохранения РФ, экспертом ВОЗ по сахарному диабету, президентом Ассоциации эндокринологов России и профессором кафедры эндокринологии Первого Московского государственного медицинского университета им. И.М. Сеченова, которые он возглавляет более 30 лет, вносит большой вклад в развитие медицинской науки, модернизацию эндокринологической и диабетологической службы в России, подготовку высококвалифицированных кадров.

Интегральными показателями эффективности эндокринологической/диабетологической службы в Российской Федерации, руководителем которой является главный специалист эндокринолог Минздрава России академик РАН И.И. Дедов, сегодня являются беспрецедентно высокие демографические показатели:

Под руководством И.И. Дедова впервые в России внедрена специализированная структурированная программа подготовки эндокринологов для регионов РФ по принципу: «от студенческой скамьи до профессорско-преподавательского состава».

Дедовым И.И. опубликовано более 750 научных трудов, из них 397 — за рубежом, в том числе 85 монографий, учебников, руководств и атласов.

Дедов И.И. — член Французской академии наук, Президент общественной организации «Российская ассоциация эндокринологов», Почетный Президент Общероссийской общественной организации инвалидов «Российская диабетическая ассоциация», объединяющей более 3,5 миллиона больных, главный редактор ряда профильных журналов.

Иван Иванович — обладатель различных наград, в том числе государственных: Ордена «Дружбы народов» (12 сентября 1994 г.), Медали «В память 850-летия Москвы» (26 февраля 1997 г.), Ордена Франции «Командора за заслуги» (05 декабря 2013г.); Ордена «Почета» (февраль 2015 г.), он также Заслуженный деятель науки РФ (25 июня 1997 г.), Полный кавалер Ордена «За заслуги перед Отечеством» (19 февраля 2001 г., 18 октября 2004 г., 2 февраля 2008 г., 26 июня 2013 г.), лауреат высшей награды Российской академии медицинских наук — «Премии и золотой медали им. Н.И. Пирогова», лауреат премии Правительства Российской Федерации в области науки и техники за создание и внедрение в практику здравоохранения Российской Федерации системы современных технологий диагностики, лечения и профилактики сахарного диабета (27 февраля 2013 г.), лауреат Государственной Премии Российской Федерации в области науки и технологий за 2017 г. за серию работ по экспериментальной и клинической эндокринологии с научным обоснованием и внедрением в здравоохранение инновационной модели персонализированной (предупредительной) медицины, и многих других.

Редакционная коллегия журнала «Проблемы эндокринологии» сердечно поздравляет своего главного редактора и желает ему здоровья и долгих плодотворных творческих лет на благо отечественной эндокринологии!

